# The Three Congresses: Their Lessons for Medicine

**Published:** 1895-10-19

**Authors:** 


### The Hospital

An Institutional Journal of

The Medical Sciences and Hospital Administration, with

Vol. XIX.—No. 472] AN EXTRA NURSING SUPPLEMENT. [Oct. 19, 1895.

### The Three Congresses: Their Lessons for Medicine.

During the closing weeks of the summer three great bodies of separated specialists of our social system have met together in annual congress. The first of these was the British Medical Association, which held its sittings just before the holidays; the second and the third were the Church Congress and the provincial gathering of the Incorporated Law Society. The three professions divide among them the greater portion of the most cultured intellect of the times. Their influence, therefore, in the making of the history and progress of the times is inevitably almost all-powerful.

But our special concern at the moment is not the influence exerted by the three professions of divinity, medicine, and law upon the community as a whole, but the object lessons furnished by the different annual gatherings, more especially those lessons which may serve for practical illumination and guidance to our own profession viewed in its entirety.

Medicine at the recent Congress of the British Medical Association appeared for the moment like the Church and the law, to have a corporate as well as an individualistic existence. But the appearance, for all practical purposes, was delusive. There was little or no attempt to shadow forth a policy for the whole profession in its relation to our public, the world, and the times. Individualism at the general meetings, almost as much as at the sectional, reigned supreme. Only in dealing with the question of midwives' registration was there any decided indication of collective action; and then practically all those most entitled to speak, and most competent to lead, were conspicuous by their absence.

Compare this attitude of mind and its practical results with the attitude and efforts of the Church Congress and the Incorporated Law Society; but especially with that of the Church Congress. One or two generations ago the Church was as limited in her outlook and as narrow and individualistic in her aims as medicine now is. She concerned herself with two questions almost exclusively—doctrine and ecclesiastical polity. The result is known to every student of history. What a contrast does a mere glance at the subjects discussed at the recent meeting of the Church Congress reveal to the philosophic mind! Socialism, Trade Unionism, the Independent Labour party, the financial position of the clergy, popular education—these and manifold other questions, having no immediate relation whatever either to doctrine or to strictly ecclesiastical polity, have occupied a great part of the energy and attention of the members at the recent congress. If it be said that the clergy were travelling beyond their sphere, and would have been well advised to leave the consideration of such questions to experts, the criticism is feeble and inept in the highest degree. The answer to it is that ever since the Church left off being a merely ecclesiastical, and became instead a great human institution, she has advanced in the esteem and affection of the public, as well as in every variety of practical efficiency, by leaps and bounds.

The object lesson here offered to medicine is as manifest as the sun at noon. Is it a legitimate object for medicine as a whole to render to the community as a whole the highest service which its scientific advancement and its improved practical skill make it so admirably competent to render? Has not medicine proved herself in every savage or half-civilised country an almost more efficient civiliser than religion itself? Even the most exclusive of ancient civilisations will fling open their gates for the admission of Western medicine, though they bar them fast against Western religion, and sometimes even against Western commerce when they can. If medical science as now understood among us is so potent a factor in modern history and development when left in the hands of a few private individuals, what might it not become if it assumed a corporate life, with corporate activity, and set itself with expansive policy to instruct, to give service, and to bear that authority in the world which the world needs, and to which proved science everywhere may so justly lay claim? The most potent missionary in the world is the doctor. Why should he be used always as an auxiliary to an ecclesiastical organisation? Why should not medicine herself spread abroad her own ample wings and brood over mankind with the blessings of physical science and of healing and guidance for the whole of our mortal life? It is our frequent contention in these pages that true medicine, scientific medicine, is yet in her earliest infancy.

Ought it not to be a chief care of all the most competent intellects among us to watch the infant's growth, and to so order affairs that she shall become great and powerful? She will then be able to play that part in the greater world which the world is inviting her to play, and which may guide social evolution towards ends hitherto unthought of, but of immeasurable importance to the whole future well-being of civilised and uncivilised man.

